# Atypical meningiomas with an immunohistochemical profile consistent with hypermetabolic or proliferative molecular groups show high mitotic index, chromosomal instability, and higher recurrence risk

**DOI:** 10.1007/s00428-023-03537-2

**Published:** 2023-04-04

**Authors:** Valeria Barresi, Serena Ammendola, Michele Simbolo, Serena Pedron, Maria Caffo, Aldo Scarpa

**Affiliations:** 1 Department of Diagnostics and Public Health, University of Verona, Policlinico G.B. Rossi, P.Le L.A. Scuro, 10, 37138 Verona, Italy; 2 Department of Biomedical and Dental Sciences and Morpho-Functional Imaging, Unit of Neurosurgery, Messina, Italy

**Keywords:** Meningioma, ACADL, MCM2, *CDKN2A*, Recurrence

## Abstract

The use of adjuvant radiotherapy is controversial for atypical meningiomas undergoing gross total resection. It has recently been proposed that meningiomas may be classified into four molecular groups (MG): immunogenic (MG1), benign *NF2*-wildtype (MG2), hypermetabolic (MG3), and proliferative (MG4). The two latter have the worst prognosis, and it has been suggested that they can be identified using ACADL and MCM2 immunostainings. We studied 55 primary atypical meningiomas, treated with gross total resection and no adjuvant therapies, to assess whether ACADL and MCM2 immuno-expression may identify patients at higher recurrence risk, thus requiring adjuvant treatments. Twelve cases resulted ACADL-/MCM2-, 9 ACADL + /MCM2-, 17 ACADL + /MCM2 + , and 17 ACADL-/MCM2 + . MCM2 + meningiomas displayed more frequent atypical features (prominent nucleoli, small cells with high nuclear-to-cytoplasmic ratio) and *CDKN2A* hemizygous deletion (HeDe) (*P* = 0.011). The immunoexpression of ACADL and/or MCM2 was significantly associated with higher mitotic index, 1p and 18q deletions, increased recurrence rate (*P* = 0.0006), and shorter recurrence-free survival (RFS) (*P* = 0.032). At multivariate analysis, carried out including ACADL/MCM2 immuno-expression, mitotic index, and *CDKN2A* HeDe as covariates, this latter resulted a significant and independent prognosticator of shorter RFS (*P* = 0.0003).

## Introduction

Meningiomas account for approximately 40% of all primary tumors in the central nervous system [1]. According to the World Health Organization (WHO), they are classified into 15 histological subtypes and three grades of malignancy [2]. The three-tiered grading system of meningiomas (CNS WHO grade) is mainly based on histopathological features [3]; however, *CDKN2A/B* homozygous deletion and *TERT* promoter (*pTERT*) mutation have been recently added as diagnostic criteria for CNS WHO grade 3 meningiomas because of their negative prognostic significance [2]. Owing to its significant correlation with disease recurrence, the CNS WHO grade of meningiomas is included in the flowcharts guiding the post-surgical treatment of these tumors. Indeed, wait and observation is indicated for patients with grade 1 meningiomas, whereas radiotherapy is indicated for patients with grade 3 meningiomas [4]. Patients with subtotal or partial resection of grade 2 meningiomas are invariably treated with adjuvant radiotherapy, whereas the post-surgical treatment of grade 2 meningiomas that underwent gross total resection is still controversial, and either radiotherapy or observation is suggested [4].

CNS WHO grade 2 meningiomas include three histological subtypes: (i) chordoid; (ii) clear cell; and (iii) atypical [2]. This latter is by far the most frequent and its diagnosis relates on the following histological features: (i) presence of 4–19 mitoses in an area of 1.6 mm^2^; and/or (ii) brain invasion; and/or (iii) at least three minor criteria among spontaneous necrosis, pattern-less architecture (sheeting), small cells with high nuclear/cytoplasmic ratio, prominent nucleoli, and hypercellularity [2]. Atypical meningiomas have a 5-year recurrence rate of approximately 50% [5]. The wide range of histological diagnostic criteria likely accounts for their heterogeneous clinical outcome. Therefore, additional factors able to stratify atypical meningiomas based on their recurrence risk would be useful to distinguish high-risk patients who could benefit from adjuvant therapy and to spare low-risk patients from adverse effects.

Recently, several studies have demonstrated that epigenetic and cytogenetic features may predict the recurrence-free survival (RFS) in patients with meningiomas [6–8]. However, these molecular assays may not be accessible to all centers because of their high costs and long turn-around time. Using a discovery cohort of 121 meningiomas (59 grade 1, 43 grade 2, and 19 grade 3) and a validation cohort of 80 meningiomas (23 grade 1, 55 grade 2, and 2 grade 3), a recent study distinguished four consensus molecular groups by the combination of DNA somatic copy-number aberrations, DNA somatic point mutations, DNA methylation, and messenger RNA abundance [9]. Each molecular group had distinctive features: immunogenic (MG1), benign *NF2*-wildtype (MG2), hypermetabolic (MG3), and proliferative (MG4) [9]. Stratification by molecular groups correlated with RFS, independently of the extent of resection, CNS WHO grade or radiotherapy, and predicted recurrence risk more accurately than CNS WHO grade and the previously described methylation-based classifications [10]. In detail, MG3 and MG4 were associated with the shortest RFS, whereas meningiomas in the MG1 group had the longest RFS [9]. Proteomic characterization showed that the different molecular groups were enriched in different proteins, namely, MG1 in S100A, MG2 in SCGN, MG3 in ACADL, and MG4 in MCM2; a subgroup analysis of 44 cases suggested that the molecular group could be predicted using immunostainings for these proteins [9]. However, the correlation between the immuno-expression of these proteins and the clinical-pathological features or recurrence probability of meningiomas remains to be analyzed.

In this study, we investigated whether the immuno-expression of ACADL and MCM2, used as surrogates of molecular groups MGM3 and MGM4, is associated with reduced RFS or with clinical, pathological and genetic features in a cohort of 55 atypical meningiomas treated with surgery and no radiotherapy.

## Materials and methods

### Cases

Fifty-five primary atypical meningiomas surgically resected from adult patients (age ≥ 18 years) between 2003 and 2018 were included in this study. The inclusion criteria were as follows: (1) atypical meningioma subtype according to WHO 2021 criteria [2]; (2) complete surgical resection (corresponding to Simpson grades 1–3 [11]); (3) no history of neurofibromatosis; (4) available information on RFS; (5) at least 24 months of follow-up for non-recurring tumors; and (6) availability of paraffin blocks.

### Ethical issues

This study was approved by Comitato Etico per la Sperimentazione Clinica delle province di Verona e Rovigo (protocol n. 40,400, 2019/07/19).

### Histological revision

We reviewed the histological slides of all cases to assess the number of mitoses per 1.6 mm^2^, brain invasion, hypercellularity, prominent nucleoli, small cells with a high nuclear-to-cytoplasmic ratio, spontaneous necrosis, and sheeting, as previously described [5, 12, 13].

### Clinical data

Information on tumor localization, extent of surgical resection, and RFS was retrieved from the clinical records and operatory registries.

Meningiomas were subdivided into three groups according to their localization: (1) meningiomas of the convexity; (2) parasagittal/tentorium; and (3) skull base.

### Immunohistochemistry

All cases were immunostained using antibodies against MCM2 (clone 1E7, dilution 1:200, Cell Signaling Technology) and ACADL (polyclonal, dilution 1: 200, Sigma).

In each case, we assessed the percentage of neoplastic cells that were positive for MCM2 and ACADL. Cases were classified as positive when > 5% of stained cells were observed.

Then, meningiomas were subdivided into three groups according to MCM2 and ACADL positivity: (i) immunohistochemical group1 (IHC-G1), including cases negative for both ACADL and MCM2 (corresponding to MG1 and MG2); (ii) IHC-G2, composed of cases positive for only ACADL (corresponding to MG3); and (iii) IHC-G3, comprising cases positive for MCM2 and either positive or negative for ACAL (corresponding to MG4).

### CDKN2A copy number variations

*CDKN2A* copy number variations were assessed using NGS (see below) in 15 meningiomas (cases 12–26) and FISH and LSI CDKN2A/CEP 9 probes (Vysis/Abbott, Molecular Europe, Wiesbaden, Germany) in 40. In detail, slides were examined using an Olympus BX61 fluorescence microscope equipped with a 100 × oil immersion objective and a triple band pass filter for simultaneous detection of Spectrum Orange, Spectrum Green, and DAPI signals. We counted 200 non-overlapping nuclei containing a minimum of 2 reference probe (CEP 9 probe) signals. *CDKN2A* homozygous deletion was defined as the presence of two green signals (control probe on chromosome 9 centromeres) and no orange signals (reference probe on *CDKN2A* locus) in at least 30% of cells.

### Next generation sequencing

In 15 meningiomas (cases 12–26), mutations and CNV in 409 genes (including *CDKN2A/B*) were previously analyzed using the Oncomine Tumor Mutational Load (TML) assay (Thermo Fisher), which covers 1.65 Mb of genomic space [8].

### Integrated molecular grading

The integrated molecular grading system proposed by Driver et al. [6] was applied to fifteen meningiomas analyzed by NGS. This is a grading system combining genetic alterations and mitotic index. In detail, one point is assigned to the presence of each genetic alteration among 1p, 3p, 4p/q, 6p/q, 10p/q, 14q, 18p/q and 19p/q deletion, and *CDKN2A/B* homozygous or heterozygous deletion; one point is assigned to mitotic index of 4–19 mitoses/1.6 mm^2^, whereas two points are assigned to mitotic index of ≥ 20 mitoses/1.6 mm^2^ [6]. Meningiomas with 0–1 point are classified as integrated grade 1, meningiomas with 2–3 points as integrated grade 2, and meningiomas with ≥ 4 points as integrated grade 3 [6].

### Statistical analyses

We used the Chi-squared test to analyze the correlations between the immunohistochemical group and the various clinico-pathological parameters (age and sex of the patients, localization, brain invasion, sheeting, hypercellularity, prominent nucleoli, spontaneous necrosis, small cells with high nuclear/cytoplasmic ratio of the tumors) or genetic features (i.e., gene mutations or CNV, chromosomal gains or losses). The Kruskal–Wallis test was used to assess whether the mitotic index differed according to the immunohistochemical group.

Finally, RFS was assessed using the Kaplan–Meier method, with the date of primary surgery as the entry data and length of survival to the detection of a recurrent tumor as the end point. The Mantel-Cox log-rank test was applied to assess the strength of association between mitotic index, brain invasion, immunohistochemical group, *CDKN2A/B* copy number, and RFS.

A probability (*P*) value less than 0.05 was considered significant. Statistical analysis was performed using the MedCalc 12.1.4.0 statistical software (MedCalc Software, Mariakerke, Belgium).

## Results

### Clinical features

**Fig. 1:**
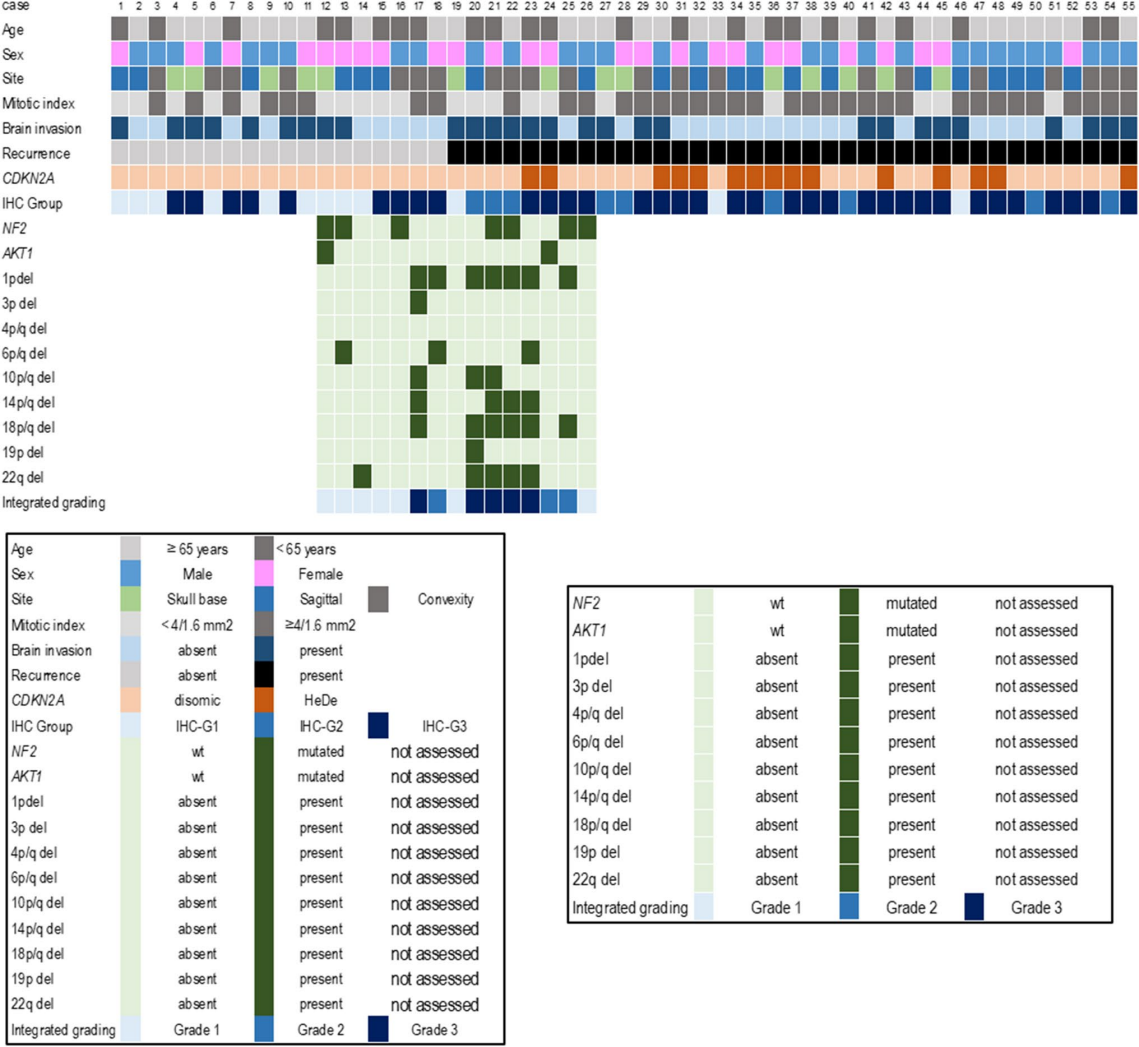
Clinical-pathological, immunohistochemical and genetic features of 55 atypical meningiomas according to WHO 2021

This study included 55 meningiomas that were surgically resected in 30 male and 25 female patients (mean age: 60 ± 13 years), with extent of surgical resection corresponding to Simpson grade 1 in 33 cases, grade 2 in 14 cases, and grade 3 in 8 (Fig. 1). Twenty-one meningiomas were located at the brain convexity, 20 were parasagittal/tentorial, and 14 were at the skull base.

Thirty-seven patients had tumor recurrence during follow-up, with an RFS length ranging between 4 and 151 months (median, 41 months). The follow-up time ranged from 32 to 105 months (median, 52 months) in patients without disease recurrence.

### Histopathological features, TERT promoter mutation, and CDKN2A copy number variations

Histologically, meningiomas were classified as atypical because of the following: (i) a mitotic index ≥ 4/1.6 mm^2^, in 35 cases; (ii) presence of brain invasion, in the absence of a high mitotic index, in 17 cases; and (iii) presence of only minor atypical criteria, in 3 cases (Fig. 1). In accordance with the inclusion criteria, none had *CDKN2A* homozygous deletion or *TERT* promoter mutations.

Fifteen cases had *CDKN2A* heterozygous deletion (HeDe) (Fig. 1).

### Mitotic index was higher in meningiomas IHC-G2 (ACADL +) and IHC-G3 (MCM2 +)

Twelve meningiomas were classified as IHC-G1 (MCM2-/ACADL-) (Fig. 1).

All cases positive for ACADL and/or MCM2 had ≥ 20% stained cells, whereas none showed a percentage of stained cells between 5 and 20%.

**Fig. 2:**
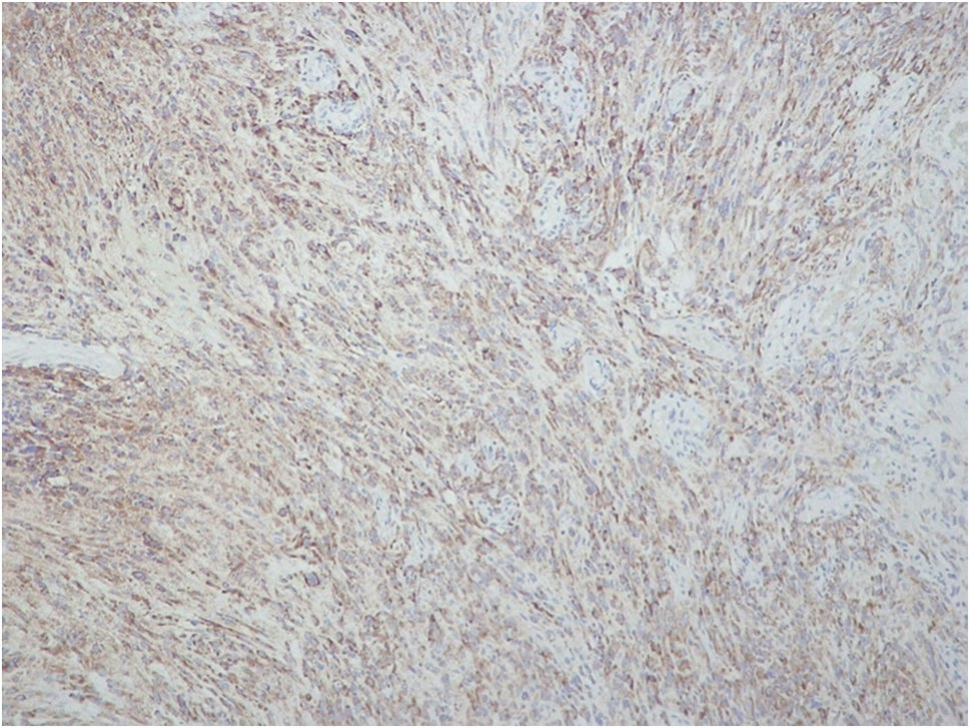
ACADL immunostaining in atypical meningioma. Diffuse cytoplasmic staining in tumor cells (original magnification, × 100)

**Fig. 3:**
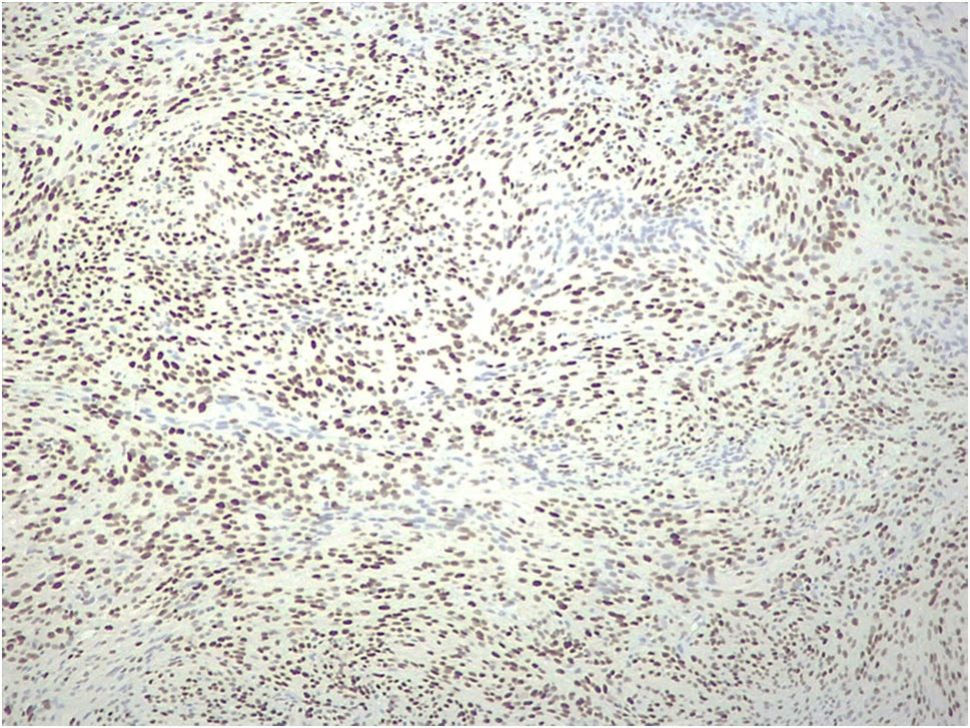
MCM2 immunostaining in atypical meningioma. Diffuse nuclear staining in tumor cells (original magnification, × 100)

Nine tumors with ACADL positivity in 20–60% of cells and lacking MCM2 immunostaining were IHC-G2 (MCM2-/ACADL +) (Fig. 2). Finally, 34 meningiomas, showing MCM2 immunostaining in 20–90% cells, were IHC-G3 (MCM2 +) (Fig. 3); 17 of these cases had concurrent ACADL immunostaining in 10–80% tumor cells.

Tumors IHC-G3 (MCM2 +) were more frequently characterized by prominent nucleoli (77% vs 64% in IHC-G2 and 33% in IHC-G1) (*P* = 0.0449) and by small cells with high nuclear/cytoplasmic ratio (66% vs. 42% and 11%) (*P* = 0.0129) (Table 1). Atypical meningiomas IHC-G2 and IHC-G3 had a significantly higher mitotic index than IHC-G1 (*P* = 0.029).

**Table 1:** Statistical correlations between immunohistochemical group (IHC-G) and clinical-pathological features or *CDKN2A* copy number in 55 atypical meningiomas according to WHO 2021

Parameter	IHC group			*P*
	IHC-G1	IHC-G2	IHC-G3	
Age				
< 65 years	7	4	21	
≥ 65 years	5	5	13	0.644
Sex				
Female	7	4	14	
Male	5	5	20	0.589
Site				
Convexity	3	5	4	
Sagittal	3	2	4	
Skull base	15	13	6	0.417
Brain invasion				
Absent	4	3	18	
Present	8	6	16	0.365
Hypercellularity				
Absent	6	5	19	
Present	6	4	15	0.937
Prominent nucleoli				
Absent	4	6	8	
Present	7	3	26	0.049
Mitotic index				
< 4/1.6 mm^2^	7	4	9	
≥ 4/1.6 mm^2^	5	5	25	0.122
Spontaneous necrosis				
Absent	8	3	11	
Present	4	6	23	0.102
Sheeting				
Absent	8	7	14	
Present	4	2	20	0.081
Small cells with high N/C ratio				
Absent	7	8	12	
Present	5	1	22	0.012
Recurrence				
Absent	9	0	9	
Present	3	9	25	0.0006
*CDKN2A*				
Disomic	12	8	20	
HeDe	0	1	14	0.011

### 1p and 18q deletion were more frequent in meningiomas IHC-G2 (ACADL +) and IHC-G3 (MCM2 +)

NGS analysis was carried out on 15 atypical meningiomas, including 4 IHCG1, 3 IHCG2, and 8 HCG3. Six cases had *NF2* mutations (1/4 IHC-G1, 2/3 IHC-G2, and 3/8 ICH-G3) and two harbored *AKT1* E17K mutation (1 IHC-G1 and in 1 IHC-G3) (Fig. 1).

The deletion of 1p (*P* = 0.0239), 10q, 14q, or 18q (*P* = 0.0125) was exclusive to ICH-G2 and ICH-G3 (Fig. 1).

### CDKN2A hemizygous deletion was more frequent in atypical meningiomas IHC-G3 (MCM2 +)

*CDKN2A* HeDe was present in 14 meningiomas ICH-G3, in one meningioma IHC-G2 and absent in all meningiomas IHC-G1 (*P* = 0.0462).

### Integrated molecular grade correlates with MCM2 and ACADL immunostaining

All four meningiomas lacking MCM2 and ACADL immunostaining (IHC-G1) were classified as integrated grade 1; all three meningiomas in IHC-G2 (ACADL + /MCM2-) were classified as integrated grade 3, whereas 5/8 tumors in IHC-G3 (MCM2 +) were classified as integrated grade 2 or 3 (*P* = 0.0166).

### Immunostaining for ACADL or MCM2 was significantly associated with tumor recurrence and shorter RFS

**Fig. 4:**
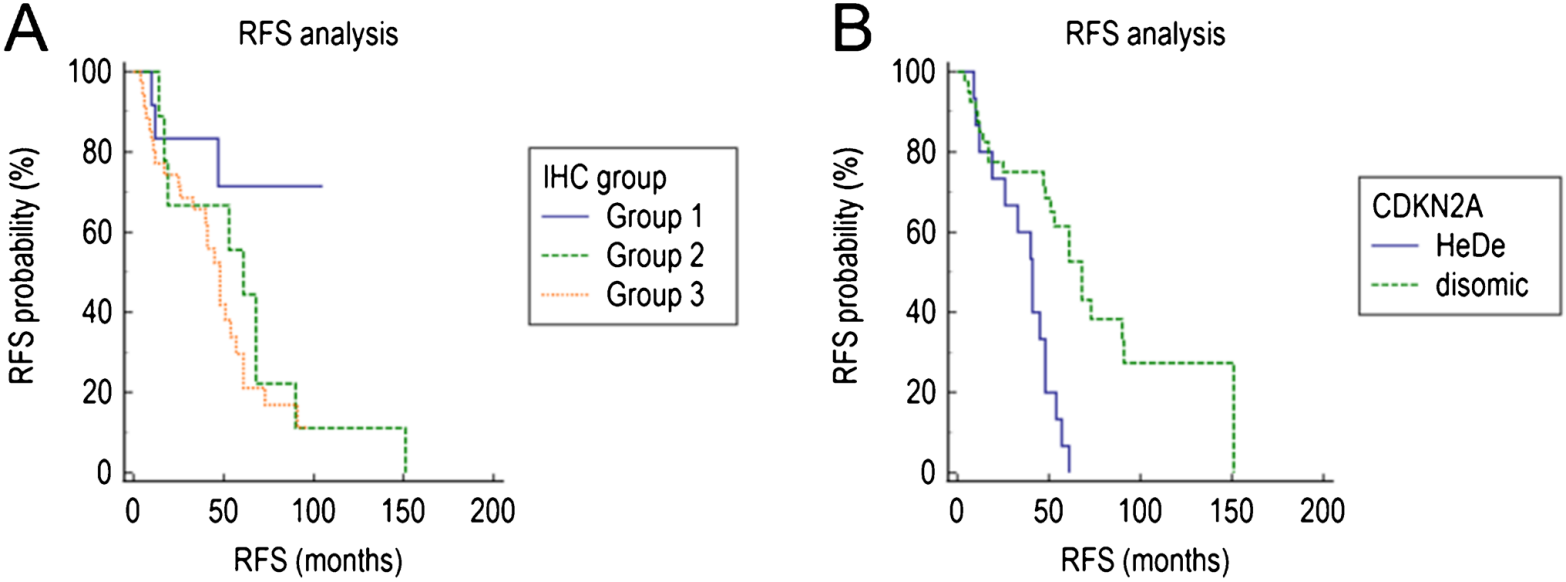
Kaplan–Meier curves showing RFS of 55 patients with atypical meningioma according to IHC group and *CDKN2A* copy number variation. **A** The RFS of patients with atypical meningioma IHC group 2 (ACADL + /MCM2-) and IHC group 3 (MCM2 +) was significantly shorter than that of patients with atypical meningiomas IHC group 1 (ACADL-/MCM2-). **B** The RFS of patients with atypical meningioma harboring *CDKN2A* hemizygous deletion (HeDe) was significantly shorter than that of patients with atypical meningiomas having disomic *CDKN2A*

Three of 12 (25%) meningiomas IHC-G1, 9/9 (100%) IHC-G2, and 25/34 (74%) IHC-G3 recurred (*P* = 0.0006) (Table 1). RFS was significantly shorter in patients with meningiomas IHC-G2 or IHC-G3 (hazard ratio, 2.3; 95% confidence interval, 1–5.1; *P* = 0.032) (Fig. 4) or having mitotic index ≥ 4/1.6 mm^2^ (hazard ratio, 2.8; 95% confidence interval, 1.4–5.5; *P* = 0.0026) or harboring *CDKN2A* HeDe (hazard ratio, 5; 95% confidence interval, 2.1–12.1; *P* = 0.0003) (Fig. 4).

Multivariate analysis (Cox regression model) was used to determine the independent effect of the IHC group, high mitotic index (≥ 4/1.6 mmq) and *CDKN2A* HeDe on RFS. A low mitotic index (< 4/1.6 mm^2^) was independently associated with longer RFS (hazard ratio, 0.3; 95% confidence interval, 0.1–0.7; *P* = 0.0082), and *CDKN2A* HeDe was independently associated with shorter RFS (hazard ratio, 3.3; 95% confidence interval, 1.5–6.9; *P* = 0.0015), whereas IHC group had no independent effect on patients RFS.

## Discussion

The treatment of patients with atypical meningiomas after gross total resection is largely debated, owing to controversial results in studies investigating the benefit of adjuvant radiotherapy in ameliorating their RFS. Controversial findings may be due to the biological heterogeneity of atypical meningiomas, which include indolent and aggressive tumors.

Nassiri et al. recently suggested that meningiomas could be classified into molecular groups, which may reflect their biological and clinical aggressiveness better than WHO histological subtypes [9]. In their cohort of 121 tumors, molecular groups were prognostically relevant even in the subgroup of 43 meningiomas classified as grade 2 according to the 2016 WHO criteria [3], with MG3 and MG4 cases having the shortest RFS (*P* = 0.037) [9]. MG3 and MG4 may be recognized using immunohistochemistry against ACADL and MCM2 [9], but whether these immunostainings may be useful to identify meningiomas with a higher recurrence risk has not been investigated.

In this study, we analyzed for the first time whether ACADL and MCM2 immunostainings, used as surrogates to determine molecular groups MG3 and MG4, could predict the recurrence risk of atypical meningiomas and to identify cases which could benefit from adjuvant treatments in clinical practice. In a cohort of 55 primary meningiomas, classified atypical according to the 2021 WHO criteria, and that underwent complete surgical resection and no adjuvant radiotherapy, immunostaining for MCM2 or ACADL in ≥ 20% tumor cells was significantly associated with recurrence and with shorter RFS, without any significant difference between tumors showing only ACADL immunostaining (corresponding to MG3) or both ACADL and MCM2 positivity (equating to MG4). These findings suggest that in routine practice atypical meningiomas can be stratified according to recurrence risk using MCM2 and ACADL immunostainings.

In accordance with the reported frequent inactivation of tumor suppressor genes in MG4 and MG3 [9], atypical meningiomas with MCM2 or ACADL immunostaining had higher mitotic counts, reflecting a high proliferative activity. In addition, they had chromosomal instability and were enriched in multiple chromosomal deletions previously associated with a higher risk of meningioma recurrence [6, 8, 14–17]. This finding is in line with the high aneuploidy found in MG3 and MG4 meningiomas, which harbor frequent losses in chromosomes 22q, 1p, 6q, 14, and 18 [9], whereas these alterations are infrequent in meningiomas of other molecular groups.

Driver et al. recently proposed a three-tiered integrated molecular grading of meningiomas based on the presence of several chromosomal deletions (1p, 3p, 4p/q, 6p/q, 10p/q, 14q, 18p/q, and 19p/q), *CDKN2A/B* deletion, and mitotic count [6]. In 527 meningiomas, they demonstrated that the overall correlation between CNS WHO and molecular integrated grading was good; however, within CNS WHO grade 2 meningiomas, the concordance with integrated grades was of only 31%, reflecting the high molecular heterogeneity of these tumors [6]. Indeed, CNS WHO grade 2 meningiomas resolved into integrated grade 1 in one-third and into integrated grade 3 in another third [6]. Although integrated molecular grading outperformed CNS WHO grading in predicting recurrence and it nicely stratified CNS WHO grade 2 meningiomas for their recurrence risk [6], its application in routine practice is limited by the high costs. In this study, we applied the integrated molecular grading to 15 atypical meningiomas (CNS WHO grade 2) according to the 2021 WHO criteria. Owing to the higher frequency of chromosomal deletions in cases with MCM2 or ACADL immunostaining, the immunohistochemical group and integrated grade were significantly correlated. Indeed, all four cases classified as integrated grade 1 lacked MCM2 or ACADL immunostaining. Therefore, these findings suggest that MCM2 and/or ACADL immunostaining may be used to identify meningiomas with high chromosomal instability and consequently a high integrated grade.

In this study, we assessed *CDKN2A* copy number variations as a requisite to exclude CNS WHO grade 3 meningioma [2]. Fifteen tumors, all positive for MCM2 or ACADL, had *CDKN2A* HeDe. Although *CDKN2A* HeDe is not currently considered as a criterion for upgrading meningiomas to CNS WHO grade 3, it was recently suggested to predict shorter RFS in patients with meningiomas [18]. Indeed, in a heterogeneous cohort of 659 meningiomas, including primary and recurrent tumors, of all three CNS WHO grades, and with either total or subtotal surgical resection, *CDKN2A/B* HeDe was found in 15 cases in association with shorter progression-free survival [18]. Our findings, obtained in a cohort of only primary atypical meningiomas that underwent total resection, further support the negative prognostic significance of *CDKN2A* HeDe in meningiomas. Indeed, this was significantly associated with unfavorable IHC groups (MCM2 + and ACADL +) and with shorter RFS, independently of these latter.

In conclusion, this is the first study to show that immunostainings for MCM2 and ACADL may be useful for identifying atypical meningiomas characterized by higher genomic instability, *CDKN2A* HeDe, higher integrated molecular grade, recurrence risk, and shorter RFS. A limitation of this study was the number of cases, although it should be emphasized that we included only primary meningiomas that underwent gross total resection. Despite the correlation with pathological and genetic features reflecting higher biological aggressiveness, MCM2/ACADL immuno-expression was not an independent prognostic factor at the multivariate RFS analysis including mitotic index and *CDKN2A* HeDe.

## Data Availability

Data will be available upon request to corresponding author,
